# Host Biology in Light of the Microbiome: Ten Principles of Holobionts and Hologenomes

**DOI:** 10.1371/journal.pbio.1002226

**Published:** 2015-08-18

**Authors:** Seth R. Bordenstein, Kevin R. Theis

**Affiliations:** 1 Department of Biological Sciences, Vanderbilt University, Nashville, Tennessee, United States of America; 2 Department of Pathology, Microbiology, and Immunology, Vanderbilt University, Nashville, Tennessee, United States of America; 3 Department of Internal Medicine, University of Michigan, Ann Arbor, Michigan, United States of America; Harvard University, UNITED STATES

## Abstract

Groundbreaking research on the universality and diversity of microorganisms is now challenging the life sciences to upgrade fundamental theories that once seemed untouchable. To fully appreciate the change that the field is now undergoing, one has to place the epochs and foundational principles of Darwin, Mendel, and the modern synthesis in light of the current advances that are enabling a new vision for the central importance of microbiology. Animals and plants are no longer heralded as autonomous entities but rather as biomolecular networks composed of the host plus its associated microbes, i.e., "holobionts." As such, their collective genomes forge a "hologenome," and models of animal and plant biology that do not account for these intergenomic associations are incomplete. Here, we integrate these concepts into historical and contemporary visions of biology and summarize a predictive and refutable framework for their evaluation. Specifically, we present ten principles that clarify and append what these concepts are and are not, explain how they both support and extend existing theory in the life sciences, and discuss their potential ramifications for the multifaceted approaches of zoology and botany. We anticipate that the conceptual and evidence-based foundation provided in this essay will serve as a roadmap for hypothesis-driven, experimentally validated research on holobionts and their hologenomes, thereby catalyzing the continued fusion of biology's subdisciplines. At a time when symbiotic microbes are recognized as fundamental to all aspects of animal and plant biology, the holobiont and hologenome concepts afford a holistic view of biological complexity that is consistent with the generally reductionist approaches of biology.

## Abstract 2

This Essay lays out ten principles that advance a unified theory of the evolution and ecology of host-associated microbiomes.

## Introduction

*“The time has come to replace the purely reductionist ‘eyes-down’ molecular perspective with a new and genuinely holistic*, *eyes-up*, *view of the living world*, *one whose primary focus is on evolution*, *emergence*, *and biology's innate complexity*.*”*—Carl Woese (2004) [1]

At the end of the 19th century, the theory of evolution via natural selection was birthed with the appreciation that individual animals and plants vary in their phenotypes and that competition at the individual level drives gradual change in the frequencies of these phenotypes [2]. From this early vantage point, fusing evolution with Mendelian genetics in the early 20th century was a seamless transition in biology, namely one based on the framework that phenotypes in the individual animal and plant are encoded by the nuclear genome under the laws of Mendelian inheritance [3–5]. In the mid-20th century, the modern synthesis grounded the nucleocentric foundation of zoology and botany in three areas: (1) the nuclear mutability and recombinogenic nature of organisms, (2) the sorting of this genetic variation by natural selection, and (3) the observations that macroevolutionary processes such as the origin of species can be explained in a manner that aligns with Mendelian genetics and microevolutionary mechanisms [6].

The foundation of the modern synthesis remains as scientifically sound today as when it was conceived. However, it is critical to recognize that microbiology was largely divorced from these early epochs in the life sciences. The modern synthesis commenced at a time when the germ theory of disease dictated the prevailing wisdom on microbes, and the molecular tools used to understand the microbial world and its influence were inferior to those available now [7–11]. The theories of gradual evolution and the modern synthesis were thus forged during periods of eukaryocentricism and nucleocentrism that did not appreciate the centrality of microbiology in zoology and botany because of limitations in perspective and technology.

Today, there is an unmistakable transformation happening in the way that life is comprehended [12–16], and it is as significant for many biologists as the modern synthesis. Animals and plants are no longer viewed as autonomous entities, but rather as "holobionts" [17–21], composed of the host plus all of its symbiotic microbes (definitions in Box 1). The term "holobiont" traces back to Lynn Margulis and refers to symbiotic associations throughout a significant portion of an organism's lifetime, with the prefix holo- derived from the Greek word *holos*, meaning whole or entire. Amid the flourishing of host microbiome studies, holobiont is now generally used to mean every macrobe and its numerous microbial associates [19,22], and the term importantly fills the gap in what to call such assemblages. Symbiotic microbes are fundamental to nearly every aspect of host form, function, and fitness, including in traits that once seemed intangible to microbiology: behavior [23–26], sociality [27–30], and the origin of species [31]. The conviction for a central role of microbiology in the life sciences has been growing exponentially, and microbial symbiosis is advancing from a subdiscipline to a central branch of knowledge in the life sciences [14,32–35].

This revelation brings forth several newly appreciated facets of the life sciences, including the testable derivation that the nuclear genome, organelles, and microbiome of holobionts comprise a hologenome [35–37]. The hologenome concept is a holistic view of genetics in which animals and plants are polygenomic entities. Thus, variation in the hologenome can lead to variation in phenotypes upon which natural selection or genetic drift can operate. While there is a rich literature on coevolutionary genomics of binary host–microbe interactions, there have been few systematic attempts to align the true complexity of the total microbiome with the modern synthesis in a way that integrates these disparate fields [38–40].

The object of this essay is to make the holobiont and hologenome concepts widely known. We clarify and append what they are and are not, explain how they are both consistent with and extend existing theory in ecology and evolutionary biology, and provide a predictive framework for evaluating them. Our goal is to provide the main conceptual foundation for future hypothesis-driven research that unifies perceived divisions among subdisciplines of biology (e.g., zoology, botany, and microbiology) and advances the postmodern synthesis that we are now experiencing [41,42]. We distill this topic with evidence-based reasoning to present the ten principles of holobionts and hologenomes (summarized in Box 1).

**Box 1. The Ten Principles of Holobionts and Their Hologenomes**

I. Holobionts and hologenomes are units of biological organization
  - Complex multicellular eukaryotes are not and have never been autonomous organisms, but rather are biological units organized from numerous microbial symbionts and their genomes.
  - Biomolecular associations between host and microbiota are more conceptually similar to an intergenomic, genotype x genotype interaction than a genotype x environment interaction.
II. Holobionts and hologenomes are not organ systems, superorganisms, or metagenomes
  - As holobionts are complex assemblages of organisms consisting of diverse microbial genomes, biology should draw a clear distinction between holobionts/hologenomes and other terms that were not intended to describe host–symbiont associations.
  - Organ systems and superorganisms are biological entities comprised of one organism's genome; metagenome means "after" or "beyond" the genome, does not intrinsically imply organismality, and obviates the fundamentals of symbiosis in the holobiont.
III. The hologenome is a comprehensive gene system
  - The hologenome consists of the nuclear genome, organelles, and microbiome.
  - Beneficial, deleterious, and neutral mutations in any of these genomic subunits underlie hologenomic variation.
IV. The hologenome concept reboots elements of Lamarckian evolution
  - Although Lamarck never imagined microbes in his theory, applying the tenets to holobionts rebirths some major aspects of Lamarckism.
  - The nuclear genome is inherited mainly within a Mendelian framework, but the microbiome is originally acquired from the environment and may become inherited.
  - Host–microbe associations can forge disequilibria via parental transfer or stable environmental transmission.
V. Hologenomic variation integrates all mechanisms of mutation
  - Every hologenome is a multiple mutant, meaning that there is variation across many individual genomes spanning the nucleus, organelles, and microbiome.
  - Base pair mutation, horizontal gene transfer, recombination, gene loss and duplication, and microbial loss and amplification are all sources of variation.
VI. Hologenomic evolution is most easily understood by equating a gene in the nuclear genome to a microbe in the microbiome
  - Evolution for both genes and symbionts is fundamentally a change in population frequency over successive generations, i.e., the fraction of holobionts carrying that particular nuclear allele or microbe.
  - Covariance of hosts and microbes in a holobiont population (i.e., community genetics) follows a theoretical continuum directly to coinheritance of gene combinations within a genome (i.e., population genetics).
  - A grand unified theory of evolutionary and ecological genetics deserves priority attention.
VII. The hologenome concept fits squarely into genetics and accommodates multilevel selection theory
  - Multilevel selection theory asserts that selection operates across multiple levels of genetic variation with phenotypic effects, from genes to hologenomes and beyond.
  - Holobionts are exclusive to hosts and their associated microbiota; different holobionts, such as a pollinator and a flower, interact with each other under standard ecological principles.
VIII. The hologenome is shaped by selection and neutrality
  - Natural selection can work to remove deleterious nuclear mutations or microbes while spreading advantageous nuclear mutations or microbes; in the absence of selection, the neutral spread of hologenomic variation through populations is an inherently stochastic process.
  - Mixed ecological models of stochastic and deterministic community assembly likely reflect natural systems, and partitioning the microbiota into stochastic versus deterministic subunits will be an important future goal of the field.
IX. Hologenomic speciation blends genetics and symbiosis
  - The Biological Species Concept was never intended to be exclusive of symbiosis, though history largely divorced the two and created unnecessary controversy.
  - Antibiotic or axenic experiments in speciation studies must be a routine, if not obligatory, set of experiments in genetic analyses of speciation for an all-inclusive understanding of the origin of species.
X. Holobionts and their hologenomes do not change the rules of evolutionary biology
  - Although the concepts redefine that which constitutes an individual animal or plant, they are not a fundamental rewriting of Darwin's and Wallace's theory of evolutionary biology.
  - Simply put, if the microbiome is a major, if not dominant, component of the DNA of a holobiont, then microbiome variation can quite naturally lead to new adaptations and speciation, just like variation in nuclear genes.

## I. Holobionts and Hologenomes Are Units of Biological Organization

Host–microbial symbioses are familiar to most biologists [14,32], yet detailed examples are often limited to very defined, often pairwise, associations [14,35,43]. The holobiont and hologenome concepts upgrade this conventional vision to encompass the vast ecological and genomic complexity of a host and its total microbiota (see Box 2). These concepts assert that macrobes are not and have never been autonomous individuals, but rather are organized biological units, i.e., holobionts, composed of hundreds to thousands of individual organisms [32,33,35,44]. Host-associated microbes have an overwhelmingly evident influence on the physiology, anatomy, behavior, reproduction, and fitness of holobionts [14,23–25,45–49]. The holobiont and hologenome concepts therefore raise the discussion of individuality [33] and organismality [50] beyond its historical perspective to a level that challenges and extends current thinking. Although there has been widespread discussion and applied success of the ecological theories underlying host–microbial interactions [51–55], the specific evolutionary principles governing these multifarious interactions remain fundamentally unexplored.

**Box 2. Terminology**

Coevolution: reciprocal evolution of interacting species

Commensalism: a relationship benefiting one party while the other is unaffected

Mutualism: a relationship benefiting both parties

Parasitism: a relationship benefiting one party to the other’s detriment

Symbiosis: two or more species living closely together in a long-term relationship

Macrobe: a eukaryotic host, most being visible by eye

Microbiota: the microbes in or on a host, including bacteria, archaea, viruses, protists, and fungi

Microbiome: the complete genetic content of the microbiota

Holobiont: a unit of biological organization composed of a host and its microbiota

Hologenome: the complete genetic content of the host genome, its organelles’ genomes, and its microbiome

Microbe flow: the exchange of microbes between holobionts

Phylosymbiosis: microbial community relationships changing in parallel with the host nuclear phylogeny

**Hologenome Concept of Evolution**

The hologenome concept of evolution was first explicitly introduced in 1994 during a symposium lecture by Richard Jefferson [56], and it was independently derived in 2007 by Eugene Rosenberg and Ilana Zilber-Rosenberg [57]. It posits that hosts and their microbiota are emergent individuals, or holobionts, that exhibit synergistic phenotypes that are subject to evolutionary forces [35–37]. Via fidelity of transmission from parents to offspring or stable acquisition of the microbiome from the environment, covariance between the host and microbiota can be established and maintained. Consequently, as with phenotypes encoded by nuclear genomes, phenotypes encoded by beneficial, deleterious, and neutral microbes in the microbiome are subject to selection and drift within holobiont populations. Genetic variation among hologenomes can arise through changes to host genomes as well as through changes to the genomes of constituent symbiotic microbes [35–37,58]. The microbiomes, and thus their encoded phenotypes, can change through differences in the relative abundances of specific symbiotic microbes, the modification of the genomes of existing resident microbes, or the incorporation of new microbial symbionts into holobionts, which can occur even within the reproductive lifetime of hosts [58]. Importantly, genetic variation in the microbiome vastly exceeds that in the host genome and accumulates much more rapidly than variation in host genomes. Therefore, given that genetic variation is the raw material upon which evolution ultimately acts, microbial sources of hologenomic variation are potential targets of evolution, and, despite its inherent complexity, biologists must consider the incorporation of the microbiome in the overall study of evolution.

A default position in modeling host and symbiont associations would be to define them as genotype-by-environment (G host x E microbiota) interactions. Another simplistic vision is that the microbiota is a phenotype encoded by the host genome [44,59–63]. These tenets are useful to a degree but merit a reexamination. Ample evidence shows that members of the microbiota are not subjected unilaterally to the host's intent but instead colonize specific hosts over other biotic or abiotic habitats [64–66]. Thus, microbes are not solely an E that succumbs to the control of a G host. They are an evolving G themselves, with their own genomes, transcriptomes, metabolomes, etc. If we took this host-centric view to its extreme opposite, then we end with the equally wrong conclusion that hosts are just an environment for microbes. Moreover, a framework for this biological organization already exists in which the genome and microbiome forge networks of G x G interactions that can in turn interact with E to potentially forge multispecific geographic mosaics of coevolution [67,68]. That is, these symbioses are best viewed as neither G × E nor G × G, but rather G × G × E. The key point here is that the biomolecular associations between host and microbiota are more conceptually similar to an intergenomic G x G network or epistasis than any alternative vision that is incapable of dealing with the nonlinear intricacies of symbioses.

Intergenomic epistasis is when genes of one species interact with specific genes in another. The interactions, and sometimes intertwining, of genomes and gene products between the host and microbiota can carry out many functions of a hologenome, such as the synthesis of essential amino acids [69], chemosynthesis [70], or metabolite production [71]. These symbiotic combinations can be transmitted across holobiont generations and are critical for the maintenance of mutualisms, homeostasis, and potential coevolutionary outcomes, such as those exemplified between the nuclear genome and mitochondria [72]. An important and appealing aspect of intergenomic epistasis is that it unifies, rather than separates, the genetics of populations and communities [73]. For instance, there is a conceptual continuum between intragenomic (or cytonuclear) interactions and intergenomic interactions between the host genome and the microbiome. The novelty and future challenge is identifying the number and types of intergenomic interactions that are ecologically and evolutionarily relevant (Box 3). This will likely require new theoretical and statistical models, e.g., from complex systems science [74–76], that may ultimately have as much bearing on contemporary and future evolutionary theory as the models underlying the modern synthesis [3,4].

**Box 3. Long-Term Inquiries of the Hologenome**

**Hologenomic homeostasis**

Although microbiota are host specific [77–84], they are often highly diverse. The same can be said of nuclear genetic variation across the genome. Thus, an important area of scholarship will be to determine the homeostatic mechanisms within hologenomes that maintain such diverse but specific host–microbe assemblies. On the surface, the challenge for selection on holobiont traits seems extraordinary given the multitude of microbes that can potentially colonize hosts. It is presumably accomplished through the host’s immune system and through competitive exclusion and antimicrobial production by members of the microbiota itself [14,35,80,85–87]. This area of inquiry, which is approachable from many disciplines, is among the primary frontiers for biologists to tackle.

**Hologenomic breadth**

It is important that we increase the comparative breadth and depth of study systems in host–microbial evolution. Much of the novelty of the hologenome concept lies in its emphasis on the integrative roles of hosts and their diverse microbiota in holobiont fitness. Well-defined host–microbial systems, in which one or two microbial partners exhibit great effect on their hosts, are tremendously valuable in elucidating the proximate aspects of symbiosis given their general tractability and ease of manipulation. However, if the hologenome concept, or any other allied theory, is robust, it must be evaluated using systems in which hosts are populated by complex microbial communities as well. While continuing to capitalize on well-defined systems, we should additionally encourage studies assessing the routes and fidelity of transgenerational host–microbial association, the strength of functional integration, and the fitness consequences of comprehensive microbiome variation in complex host–microbial systems. This will require concomitant advances in multi-omics analytical techniques and complex systems modeling, thereby catalyzing transdisciplinary discoveries in the process.

**Population and community genetics**

To determine if evolutionary changes at the hologenomic level are indeed concordant with evolutionary changes at the nuclear level, there are a handful of critical questions that must be answered across a broad swath of animal and plant clades. How stable is the interspecific covariance, or correlation, between a host and its microbiota and their interacting genes? How consistent is microbial transmission from one holobiont generation to the next? Is genetic disequilibria between host and microbial genes strong enough for evolution to drive covariance and changes in their frequencies over multiple holobiont generations? How much intergenomic epistasis occurs in the hologenome such that one nuclear allele's effect on a trait depends on the state of another microbial allele? Although these inquiries are formidable, they are unquestionably within the realm of population and community genetics approaches.

The debatable and testable issue of the hologenome is whether nuclear genes and microbes are coinherited to a degree that evolution can operate on their interaction. Coinheritance of hologenomic interactions can occur either by vertical transmission via internal (e.g., transovarial) or external (e.g., breast milk) transfer mechanisms or through stable symbioses acquired faithfully from the environment. We discuss these crucial transmission mechanisms further in principle IV.

## II. Holobionts and Hologenomes Are Not Organ Systems, Superorganisms, or Metagenomes

There appears to be a considerable number of misplaced characterizations and colloquialisms used to refer to host-microbiota symbioses, and these misnomers can potentially act as impasses to new advances. In this section, we adapt and append the lucid clarifications previously noted in *The Hologenome Concept* [35]. First, neither the holobiont nor the microbiota should be labeled as an organ system or organ, despite frequent uses in the popular media and scientific literature [88–90]. An organ or organ system is strictly composed of cells from the same genome that perform one or more specific functions. In contrast, the microbiota is a multispecies consortia of cells with many genomes that can contribute to multiple functions throughout the body. Second, the holobiont is not a superorganism. This term is exclusively used in the context of an assembly of multiple individuals from the same species, such as in colony-forming ants, wasps, bees, and termites [91,92]. The holobiont is instead composed of multiple domains of life, as well as viruses. Finally, the term metagenome is not equivalent to hologenome. Metagenome refers to the sum of genetic information from an environmental sample and was first used in this context to describe the collective genomes of soil microbes [93]. Meta means “after” or “beyond” in Greek. Equating an environmental metagenome to a host's hologenome obviates the fundamentals of symbiosis in the holobiont. Consider the thought exercise of removing the bacterial metagenome from soil and hosts. In nature, soil would persist, but the host would not. While we understand that metagenomics will not be restrained by any one definition, we and others also recognize the salience of clear definitions in this nascent field, particularly ones that distinguish the metagenome "beyond" the soil from the hologenome that encompasses the "whole" collection of genomes in a holobiont. To summarize, biology can and should draw a clear distinction between the hologenome and other terms that were never intended to describe host-symbiont associations, including organ, superorganism, and metagenome.

## III. The Hologenome Is a Comprehensive Gene System

The geneticist Sewall Wright stated that "selection, whether in mortality, mating or fecundity, applies to the organism as a whole and thus to the effects of the entire gene system rather than to single genes" [94]. In other words, selection operates on phenotypes encoded by the organism's underlying gene system. In this light, the hologenome is the entire gene system of the holobiont, including elements of the nuclear genome, organelles, and microbiome that increase fitness, decrease fitness, or do not affect fitness at all. Within these genomic subunits, mutations are constantly arising at their own finite rates. In the nuclear genome, selection fixes favorable variants and purges the deleterious ones, or "selfish" genes can spread to enhance their own fitness. In the microbiome, selection favors the spread of beneficial microbes involved in nutrition, defense, or reproduction [20], while pathogenic microbes are either purged by holobiont selection or the pathogens deploy adaptations such as reproductive distortions to enhance their selfish transmission to the next generation [95,96]. Moreover, neutral mutations in the nuclear genome can drift to fixation or extinction across generations, as do microbes without any fitness consequences. Thus, nuclear genes with adaptive, deleterious, and neutral mutations that change their frequencies in a holobiont population are generally analogous to beneficial, parasitic, or neutral microbes that also change their frequencies in a holobiont population. How these entities change their frequencies can of course vary with transmission mode, and we address similarities and differences below. Also, classifying microbes at just one end of the symbiotic spectrum pigeonholes the reality that microbial symbioses can be pleiotropic or context-dependent. These varied evolutionary forces can sufficiently explain why animal and plant holobionts harbor species-specific microbial communities that are segregated into their own limited supply of hologenomic variability [31,35].

If hologenomic variation underscores fitness differences, then manipulating the total microbiota will alter host fitness, and therefore germ-free, gnotobiotic, and transbiotic (i.e., populated by an atypical microbiota) hosts will exhibit reduced fitness compared to wild-type and conventionalized hosts. Such predictions need assessment among a broad phylogenetic range of hosts, but ample evidence already exists. For example, in hemipteran insects, germ-free and interspecific gut microbiota cause a decrease in survivorship and delayed development in comparison to control or conventionalized species [97,98], and mice with human gut microbiota have a global immunodeficiency including less T cell proliferation and increased susceptibility to enteric infection [99]. Moreover, interspecific hybridizations can lead to a breakdown in hologenomic interactions within species [100,101].

## IV. The Hologenome Concept Reboots Elements of Lamarckian Evolution

The nuclear genome is inherited mainly within a Mendelian framework, and the microbiome is presumed to be mostly acquired from the environment or inherited uniparentally [102–106]. Whether these different transmission modes can be unified into a coherent evolutionary theory depends in part on whether dynamics between host and symbiont genes in the hologenome (e.g., intergenomic epistasis and coinheritance) are similar to dynamics between genes in the same nuclear genome [107]. In considering how genome-microbiome disequilibria, i.e., statistical associations of covariance, among hologenomes could arise, let's begin with the simplistic assumption that hologenomic change commences with Lamarck's fundamental evolutionary theory [58], generally defined as inheritance of acquired characteristics. Although evolution has had a long and tenuous history with Lamarckism [108,109], it is time to integrate it to a degree alongside Darwinism in light of modern advancements. Consider the cases of mitochondria and insect endosymbionts as textbook examples of bacteria that were once acquired from the environment during an organism's lifetime but now are vertically inherited over generations. It follows that the principal tenets of Lamarckism are operational in the origins of intimate symbioses: holobionts can gain symbiotic traits through environmental acquisition of microbes, and holobionts can potentially pass these traits on to the next generation via vertical transmission. Although Lamarck never imagined microbes in his theory, applying the tenets to holobionts rebirths Lamarckism, as some have duly noted [35,58,110].

Once new host-microbe associations are established, they can be maintained in disequilibria via vertical or stable environmental transmission [35,111]. Persuasive evidence is thoroughly reviewed elsewhere [35,105,106]. The more generations for which hologenomic disequilibrium is maintained, and the more significant the variants’ fitness effects, the more likely it is that selection will operate on them to drive changes in their frequencies. While some microbes are vertically transmitted and thus fit seamlessly into current population genetic theory, other microbes are generally not assumed to be vertically transmitted sensu strictu from one generation to the next, though we need to delve much deeper into these areas. Some fraction of the microbiota may also be acquired in a stable manner from the environment each generation, while the other fraction may be more permissive across holobiont generations. It is also important to note that vertically transmitted microbes do not have to remain present through a holobiont's lifetime nor comprise a major fraction of the microbiota to play out their evolutionary role. For instance, they may come and go across a lifetime or body site to be vertically transmitted and may also shape critical microbial successions that occur during development. Lastly, although the relationships between hosts and microbial symbionts could be fragile when there is deviation from vertical transmission [112], such concerns are no more or less valid than those for gene-gene interactions within a nuclear genome that can be broken up by recombination [107]. A critical point is that covariance of genes within and between genomes is fundamentally similar, and associations can be reinforced by population structuring and symbiont-host epistasis.

If portions of the microbiome are transmitted with fidelity across holobiont generations or stably acquired from the environment, we expect at least three types of evolutionary outcomes. First, offsprings’ microbiota and/or microbiomes should be more similar to those of the respective organs of their parents at a similar age than to those of other unrelated adults in the population. Second, for inherited microbes, experimentally tagged (e.g., genetically labeled [113,114]) microbes in adult organs should appear in the respective organs of their offspring at a similar age more often than their offsprings’ peers. Third, host immune systems, morphological structures, and/or behavioral repertoires should include mechanisms to promote the effective transmission of beneficial microbes from parents to offspring. Some illustrative model systems are already well developed [115–117]. Broadly evaluating immunological and behavioral mechanisms for transmission of microbial partners across holobiont generations should be a future research priority [23,118].

## V. Hologenomic Variation Integrates All Mechanisms of Mutation

Every hologenome is a multiple mutant, meaning that there is variation across many individual genomes spanning the nucleus, organelles, and microbiome. Without this variation, there can of course be no evolutionary change in a population of holobionts. Random nucleotide changes are the most obvious source of variation in the hologenome, followed by recombination within and between chromosomes, horizontal gene transfer within and between holobionts, and duplications/losses of gene regions. These changes can occur in any portion of the hologenome, so there is potential for immense genetic diversity across the entire gene network.

Features of the microbiome such as fluctuations in microbial abundances within holobionts are also sources of variation [35]. Indeed, they are akin to gene duplication events driving changes in a nuclear gene's abundance. For instance, the same microbial lineage that occurs at different relative abundances in two otherwise genetically identical holobionts could have different functional consequences that selection can act upon. The most obvious illustration is when a microbe operates as a commensal when rare but as a pathogen when relatively abundant [119–122]. Here, the fitness of the holobiont can change dramatically. Moreover, since no two holobionts develop in exactly duplicate environments, there can be continuous establishment and evolution of holobiont-specific microbes at different relative abundances that may drive evolutionary change.

Any analysis of holobionts and their hologenomes must also account for the multiple generations that microbes experience within the host's single generation. These differences in generation time are not fatal to the concepts, but they likely affect evolutionary outcomes of the symbiosis. For example, the propensity for symbiosis to drive molecular complexity is now a foundational premise [123], such as in obligate symbionts (with their own generation times) supplementing the missing nutrients in the inadequate diets of thousands of holobiont species spanning cicadas, bedbugs, and aphids [124]. In cicadas, the case is so extreme that genomic and cellular complexity has increased even in the absence of new symbionts via symbiotic heteroplasmy [125]. Notably, even nuclear genomes of mammalian species including humans, nonhuman primates, rodents, and elephants increase in complexity via microbial symbiosis and independent gene transfer events from virus-derived elements [126]. Similarly in *Drosophila melanogaster*, viral sequences are endogenized adjacent to retrotransposon DNA, and when transcribed, the RNA is altered by the RNA interference (RNAi) machinery and used as part of the immune system to combat lethal viral infections [127].

## VI. Hologenomic Evolution Is Most Easily Understood by Equating a Gene in the Nuclear Genome to a Microbe in the Microbiome

Is the hologenome concept refutable? We believe it is and suggest the implementation of the following litmus test: are evolutionary changes at the hologenomic level fundamentally in conflict with evolutionary changes at the nuclear gene level? Or to put it more simply, how is the evolution of a nuclear gene any different than the evolution of a microbial symbiont in a holobiont population? While the strength of selection, levels of genetic variation, and transmission strategies differ between nuclear genes and microbes, they also vary among different types of genes in the same nuclear genome. The important point is that evolution for both genes and symbionts is fundamentally a change in frequency over successive generations, i.e., the fraction of holobionts carrying that particular nuclear allele or microbe. Therefore, there is no intellectual disparity in contemplating the spread of a nuclear gene as akin to the spread of a microbe through a holobiont population. Hologenomic evolution occurs when one whole animal or plant, i.e., holobiont, leaves a different number of reproducing progeny than another, thereby changing the frequencies of their associated genes in the next generation.

Covariance of hosts and microbes (i.e., community genetics) in a holobiont population is important to this discussion as it follows a theoretical continuum directly to coinheritance of gene combinations within a genome (i.e., population genetics) [73]. The parameter Θ is useful here as it is the degree of coinheritance of polygenic or hologenomic combinations. When Θ is low because of recombination of nuclear genes or random horizontal transmission between hosts and microbes, there is little heritability and therefore selection will have little effect on the combinations. When Θ is high because of linkage disequilibria in the same genome or covariance of hosts and microbes, then evolution will operate on the combinations in a manner similar to as if they were single genes. Intermediate levels of Θ are likely to reflect natural systems and the limits of inference.

Historically, models of evolution have not properly accounted for genetically complex traits, even in the nuclear genome, because multiple genetic signals underlying phenotypes are more diffuse [128]. Yet, high-throughput sequencing techniques have enabled genome-wide association studies that map many small-effect alleles associated with phenotypic variations. Similarly in the microbial sciences, it is becoming increasingly appreciated that animal and plant holobionts are multispecies modules in which polygenic and complex systems theories of phenotypic variation are needed to identify signals of hologenomic functions and evolutionary events. These questions and ideas are an important priority for future research and emphasize the theoretical and genetic continuum between polygenic traits in the nuclear genome and host–microbe interactions in the hologenome [107]. Thus, holistic theoretical and experimental models deserve priority attention in which the genes and organisms underlying hologenomic traits vary in their inheritance mode, heritability for the traits, and linkage disequilibria. Moreover, a hologenomic framework may lead to resolving part of the missing heritability problem for complex traits that are attributable to both the nuclear genome and the microbiome.

## VII. The Hologenome Concept Fits Squarely into Genetics and Accommodates Multilevel Selection Theory

Multilevel selection theory asserts that selection operates across multiple levels of genetic variation with phenotypic effects [129], i.e., genes, chromosomes, genomes, cytonuclear interactions, groups, symbionts, communities, species, etc. Evolution as a change in allelic frequencies undoubtedly applies as all these entities have mutations that could lead to phenotypic variation. Under a framework in which evolutionary individuality includes hologenomic networks, fitness differences can arise not only from nuclear or cytoplasmic mutations, but also from host–microbe associations. Therefore, both evolutionary and ecosystem adaptive change are relevant to the study of fitness. Indeed, the proposal of an "eco-evolutionary" framework, the interplay between evolutionary and ecological dynamics [130–134], is worthy of serious attention as biology evolves to handle the combinatorial nature of hologenomic units of evolution.

A limitation of scaling the so-called "individual" unit of evolution to a holobiont is that biologists may ask: where does the multiorganismal assembly of the holobiont end? Does it proceed ad infinitum? Interactions between symbiotic microbes and their hosts make sense, but should interacting holobionts themselves be considered part of the same inclusive holobiont? For instance, do the genomes of an insect pollinator and flower constitute a hologenome? The answer here is not complex. Holobionts and their hologenomes are exclusive to the hosts and their associated microbiota. Different holobionts, such as the aforementioned pollinator and flower, clearly interact, but these interactions are not new to biology, as they form the basis for all past and present ecological investigations [135,136]. They are simply holobionts themselves interacting with each other.

## VIII. The Hologenome Is Shaped by Selection and Neutrality

Natural selection acts on holobiont phenotypes encoded by any potential source of variation in the hologenome. As previously introduced in principle III, selection can work to remove deleterious nuclear mutations or microbes while spreading advantageous nuclear mutations or microbes. In the absence of selection or when variants are selectively equivalent, the neutral spread of hologenomic variation through populations is an inherently stochastic process. For instance, many microbes could replace each other over holobiont generations because of redundant functions [137]. This may explain why animals generally have an evident core microbiota at higher taxonomic levels, i.e., phylum, but not at lower levels, i.e., species [85,138–140]. Neutral evolution in the nuclear genome can also occur when nuclear allelic variants with the same function replace each other. It is crucial to remember that neutrality does not necessarily mean that variants are functionless. Functional constraints and therefore negative selection are consistent with the neutral theory. Thus, both natural selection and neutral evolution can be seen as part of the spectrum of evolutionary possibilities operating on the hologenome.

Beyond this evolutionary framework, various ecological theories of community assembly are also relevant for determining whether the microbiome is constructed stochastically or deterministically [52,141–143]. Neutral theories of ecology emphasize the role of chance in community assembly because ecological drift and random dispersal can affect which microbial species inhabit a holobiont. If microbial community structure and dynamics are primarily stochastic, then community composition should not differ from expectations based on random community assembly models [142,143]. In contrast, if the host-associated microbiota is deterministically assembled, i.e., by host-microbiota interactions, then its composition will consistently deviate from neutral expectations. Mixed models of stochastic and deterministic community assembly likely reflect natural systems, and partitioning the microbiota into stochastic versus deterministic subunits will be an important future goal of the field.

What experiments can detect non-neutral dynamics in the hologenome? Consider the following scenario involving a genus of closely related holobiont species reared in an unbiased manner. As horizontal transmission is the presumed main mode of acquisition for the microbiota [35,106], the microbial community is not a priori expected to change in parallel with the host nuclear phylogeny unless hologenomic interactions generate specificity and codivergence between the genome and microbiome—a process that we previously termed “phylosymbiosis” [100]. Phylosymbiosis does not assume that microbial communities are stable or vertically transmitted from generation to generation. Instead, phylosymbiosis predicts that for each generation, intraspecific microbial communities are more similar to each other than to interspecific microbial communities, and the levels of genetic divergence between hosts will associate with the relative differences between their microbial communities, yielding phylosymbiotic concordance. Thus, given a genus of closely related animal or plant species, the host and microbiota can either assemble (1) randomly by stochastic processes without concordant relationships or (2) phylosymbiotically by deterministic processes in which the relationships of the microbiota are concordant with ancestry. At present, evidence for phylosymbiosis under diet-controlled regimes exists only in *Nasonia* [144] and *Hydra* [145], but the pattern also occurs in wild populations of sponges [146], ants [147], and apes [148,149]. Testing null models of population genetics and ecology for the hologenome will require the application of current and new statistical tests to distinguish selection from neutrality at both evolutionary and ecological scales.

## IX. Hologenomic Speciation Blends Genetics and Symbiosis

The Biological Species Concept [150] importantly offers a research program to explain the origin of species—namely, the evolution of barriers to interbreeding, i.e., reproductive isolation. In the absence of reproductive isolation and unlimited interbreeding between holobionts, complete gene flow and "microbe flow," a term we introduce here to denote the exchange of microbes between holobionts, can act as cohesive forces merging holobiont populations back into a cohesive group. In contrast, isolating mechanisms such as ecological isolation, mate discrimination, and hybrid incompatibilities may serve as traits that drive holobiont populations into incipient species with unique sets of hologenomic associations [151–154].

Despite the century-long paradigm of studying speciation genes in nuclear genomes of model systems, the Biological Species Concept was never intended to be exclusive of speciation symbionts [31,155]. Indeed, Theodosius Dobzhansky's graduate student Lee Ehrman pioneered studies of symbiosis to explain Haldane's rule in *Drosophila* [156]. Today, there are numerous holobiont systems wherein speciation microbes have been identified [31]. In fact, the number is similar in scope to the quantity of known speciation genes, and we ponder how many genetically mapped traits involved in intrinsic isolation could be "cured" if the microbiome was removed. Antibiotic or axenic experiments in speciation studies must be a routine, if not obligatory, set of experiments in genetic analyses of speciation. The simple ability to rear closely related animal species and their hybrids free of bacteria and then to inoculate bacteria back into axenic animals permits a gain-and-loss investigation of whether microorganisms underlie any isolating barrier between holobiont species. The study of hologenomic speciation is no longer optional—it is a necessary frontier that must be traversed for an all-inclusive understanding of the origin of species (Box 4).

**Box 4. Hologenomic Speciation**

Animal and plant species do not arise exclusively from divergence in their nuclear genomes [31]. Instead, symbiotic and nuclear genetic components can cause isolation barriers that influence the evolution of new animal and plant species. We argue that a combinatorial nature of hologenomic speciation is a far more accurate vision of speciation than has been traditionally recognized. Just as a speciation geneticist might inquire how many genes cause reproductive isolation and identify their functions [157,158], a speciation microbiologist would inquire how many host-associated microbes cause reproductive isolation and determine what kinds of microbes they are [31]. By simultaneously pursuing both sets of the questions rather than one or the other as is usually done, speciation biologists can achieve a unified theory of the Biological Species Concept that fuses symbiosis and Mendelian genetics. For instance, in the case of mushroom-feeding *Drosophila* flies or *Nasonia* parasitoid wasps, both symbiotic and nuclear genetic components combine to cause nearly complete reproductive isolation between young species [100,159,160]. All that matters is that the hologenomic components, the collection of host, organelle, and microbial DNA, function in isolation barriers.

Pioneering work on symbiont-induced speciation traces back to Lee Ehrman and her studies of infectious hybrid sterility between subspecies of *D*. *paulistorum* [161]. The bacterial infections in the testes were later identified as beneficial *Wolbachia* within the subspecies that functionally breakdown in hybrids [162], similar to how adaptive nuclear genes within species can also breakdown in hybrids. Another salient example is the evolution of *Wolbachia*-induced F_1_ hybrid inviability in the incipient stages of speciation between closely related *Nasonia* species [160,163]. Symbiont-induced, behavioral barriers to reproduction occur as well. For instance, variation in the gut microbiota, and consequently host odor profiles, causes premating isolation between strains of *D*. *melanogaster* [152].

Speciation genetic experiments are classically designed to rule in nuclear genes by mapping traits to chromosomal regions, but they fail to assess microbes as causes of reproductive isolation. As a result, the significance of microbial-induced isolation has undoubtedly been underassessed. We propose that microbe-free experiments be universally implemented in speciation studies to upgrade this narrow approach. By way of illustration, one of the best-studied genes involved in *Drosophila* adaptive evolution and hybrid inviability, *Nup96* [164,165], encodes a component of the nuclear pore complex that is hijacked by viruses to breach the nucleus [166]. Thus, mapping speciation genes to nuclear chromosomes is not evidence against hologenomic speciation sensu strictu, as some have previously noted. Rather, speciation genes in the nucleus may be half of the story as they often interact with the microbiota to cause reproductive isolation. This precedent is evident in *Nasonia* in which quantitative trait loci that associate with F_2_ hybrid lethality are contingent on the presence of the *Nasonia* gut microbiota [100].

The large and integral role of immune genes on reproductive isolation in both animals and plants has been previously termed the "Large Immune Effect” [31]. The immune system rapidly evolves to handle the resident microbiota of the holobiont, namely a finite subset of host-associated microbes spanning mutualists, pathogens, and commensals. For instance, molecular population genetic studies demonstrate in *Drosophila*, humans, and chimps that defense and immunity genes evolve more rapidly and are under more positive selection than the rest of the genome [167–169]. Immunity genes can also be preferentially misexpressed (i.e., either an increase or decrease in levels of expression compared to the parental expression) in some hybrids, suggesting that the genes subject to high rates of positive selection within species are also the ones likely to be aberrantly expressed in hybrids. For example, in the hybridization of *D*. *melanogaster* and *D*. *simulans*, we previously calculated that 93% of the immune genes were differentially expressed relative to the nonhybrid controls, compared with 57% of the nonimmune genes [31,170]. Hybrid autoimmunity is a frequent occurrence in plants as well [171,172]. Immune gene breakdowns in hybrids are likely windows into speciation by symbiosis and the hologenomic complexities maintaining host–microbe homeostasis. Indeed, in a recent study of the house mouse hybrid zone, hybrids displayed numerous differences in their microbiota, increased gut pathology, and altered immune gene expression [173]. Cases of accelerated rates of immune system evolution and positive selection within species coupled with aberrant immune gene function and gut microbiota in postmating reproductive isolation are verifications for hologenomic speciation. Moreover, the microbiota itself is now recognized as essential in the training and function of the holobiont immune system [47], including the remarkable possibility that mucus-associated bacteriophages operate as part of the adaptive and innate antimicrobial immune system [174]. Finally, the study of microorganisms associated with disease agents is poised to greatly impact our knowledge and therapeutic treatments of infectious diseases [175]. Collectively, these efforts and views should lead to deeper insights into host–microbial relationships and provide exciting new opportunities for the study of the origin of animal and plant species.

## X. Holobionts and Their Hologenomes Do Not Change the Rules of Evolutionary Biology

It is possible that preconceptions about how evolution works might cause some to think that the hologenome concept changes the way they understand evolutionary biology. However, there is no fundamental rewriting of Darwin's and Wallace's theory of evolutionary biology involved in this concept. Like single nucleotide mutations, acquisition of new symbionts births raw genetic variation that evolution can operate on. If one looks at the host-associated microbiome as a major, if not dominant, component of the DNA of an animal or plant, then transmissible changes in the microbiome can quite naturally lead to new adaptations and speciation just like changes in nuclear genes. We adhere to this general view and invite the community to consider an expansive but not revolutionary extension of evolutionary genetics in light of the heritable [60,78] and inherited microbiome [103,105]. In the perennial debate about whether evolutionary biology needs a rethink [42], it has already been noted that "this expansion of evolutionary biology does not denigrate Darwin in the least but rather emphasizes the fertility of his ideas" [41].

## Conclusion

At a time when symbiotic microbes are recognized as fundamental to nearly all aspects of animal and plant biology, the holobiont and hologenome concepts afford holistic, eyes-up views of the multicellular eukaryotic world that are consistent with the generally reductionist approaches of evolutionary biology. Rather than transforming evolutionary thought, the hologenome concept develops it in a substantive and timely way. From a specific standpoint, the holobiont and hologenome concepts redefine that which constitutes an individual animal or plant by asserting that hosts and their symbiotic microbes are complex units of biological organization upon which ecology and evolution can act. From a general standpoint, the concepts assert that macrobe evolution has been driven by both population and community genetics and that symbiotic microbes and nuclear genes hold equivalent significance in the origin of new holobiont species. Like all good scientific theories, the concepts are subject to refutation, and in this essay, we have explained how they can be empirically and experimentally falsified. We anticipate that the conceptual foundation provided in this essay will serve as a roadmap for hypothesis-driven, experimentally validated research on holobionts and their hologenomes.
